# A Review of Sampling and Monitoring Methods for Beneficial Arthropods in Agroecosystems

**DOI:** 10.3390/insects9040170

**Published:** 2018-11-23

**Authors:** Kenneth W. McCravy

**Affiliations:** Department of Biological Sciences, Western Illinois University, 1 University Circle, Macomb, IL 61455, USA; KW-McCravy@wiu.edu; Tel.: +1-309-298-2160

**Keywords:** sampling methodology, bee monitoring, beneficial arthropods, natural enemy monitoring, vane traps, Malaise traps, bowl traps, pitfall traps, insect netting, epigeic arthropod sampling

## Abstract

Beneficial arthropods provide many important ecosystem services. In agroecosystems, pollination and control of crop pests provide benefits worth billions of dollars annually. Effective sampling and monitoring of these beneficial arthropods is essential for ensuring their short- and long-term viability and effectiveness. There are numerous methods available for sampling beneficial arthropods in a variety of habitats, and these methods can vary in efficiency and effectiveness. In this paper I review active and passive sampling methods for non-*Apis* bees and arthropod natural enemies of agricultural pests, including methods for sampling flying insects, arthropods on vegetation and in soil and litter environments, and estimation of predation and parasitism rates. Sample sizes, lethal sampling, and the potential usefulness of bycatch are also discussed.

## 1. Introduction

To sustainably use the Earth’s resources for our benefit, it is essential that we understand the ecology of human-altered systems and the organisms that inhabit them. Agroecosystems include agricultural activities plus living and nonliving components that interact with these activities in a variety of ways. Beneficial arthropods, such as pollinators of crops and natural enemies of arthropod pests and weeds, play important roles in the economic and ecological success of agroecosystems. These arthropod-mediated ecosystem services (AMES) increase agroecosystem productivity and sustainability by enhancing crop yields and reducing reliance on pesticides. The annual combined value of pollination services by non-*Apis* bees and pest control provided by insect predators and parasitoids has been estimated at $7.5 billion annually in the USA [1].

Land use practices can have adverse effects on these beneficial arthropods. Intensive agriculture can negatively affect bee diversity and pollination, and there is evidence of causal connections between decline of pollinators and decline of plants to which they are functionally linked [2]. Such adverse effects can be mitigated if undisturbed pollinator habitat is available [3,4]. Likewise, agroecosystems are often unfavorable environments for arthropod natural enemies of agricultural pests, but efforts such as habitat enhancement to conserve these natural enemies can provide important control of these pests [5]. For bees and natural enemies of insect pests, the ability to effectively assess and monitor their abundance and diversity, and potential effects of land use practices on them, is of vital importance. A variety of sampling methods is available, but these methods can be biased and vary widely in performance [6,7,8,9,10,11]. Some may provide absolute estimates of abundance (density, or numbers per unit area), but most provide relative estimates that are related to the sampling method being used (e.g., numbers per trap per day, numbers per hour of aerial netting, etc.). In this paper I present a brief overview of these beneficial arthropods and their importance, and some general concerns in regard to sampling and monitoring of these organisms. I then review the major sampling and monitoring methods employed to assess these organisms, and the advantages and limitations of these methods. It is hoped that this paper will provide entomologists and crop protection specialists with a useful overview of sampling methods available and will encourage awareness and further research and conservation of these important arthropods.

## 2. Non-*Apis* Bees

**Figure 1:**
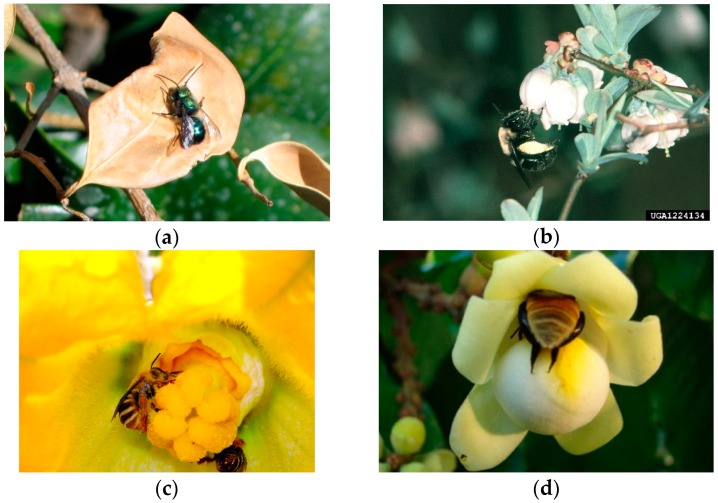
Some economically important non-*Apis* bees: (**a**) Blue orchard bee, *Osmia lignaria* Say, a common orchard pollinator; (**b**) southeastern blueberry bee, *Habropoda laboriosa* (Fabricius), an early spring pollinator of rabbit-eye blueberry, *Vaccinium virgatum* Aiton; (**c**) *Peponapis pruinosa* Say, a pollinator of *Cucurbita* spp.; and (**d**) the orchid bee *Eulaema mocsaryi* Friese, a pollinator of Brazil nut, *Bertholletia excelsa* Humb. & Bonpl. Attributions: (**a**) Robert Engelhardt [27]; (**b**) Jerry A. Payne, USDA Agricultural Research Service [28]; (**c**) Jim Cane, USDA Agricultural Research Service [29]; and (**d**) M. C. Cavalcante, F. F. Oliveira, M. M. Maués, and B. M. Freitas [30].

The total value of pollination services worldwide has been estimated at $217 billion [12]. Approximately 78 to 94% of flowering plants, and most major agricultural crops, are pollinated by animals, with bees being the predominant pollinating taxon [1,4,13]. While managed honey bee (*Apis mellifera* L.) hives are used in much of the world, availability of these hives is limited in many regions [14,15]. Honey bees are the most commonly used pollinators in large-scale commercial agriculture, but non-*Apis* bees (Figure 1) are also important in crop pollination, especially in more complex agroecosystems and diverse landscapes [16]. In the USA, non-*Apis* bees comprise almost 25% of crop pollination, at a value estimated at $3.07 billion annually [1]. High diversity of bees can enhance pollinator functional complimentarity and ensure pollination function [17,18,19]. Pollination can contribute to the nutritional value, shelf life, and quality of food crops as well [20,21].

Some studies have documented declines of non-*Apis* bees in specific geographic locations [22,23,24], but large-scale, long-term, and statistically robust monitoring programs that yield data on population changes are badly needed. To document future changes in bee diversity and evaluate the effectiveness of mitigation efforts, reliable sampling methods are required. A variety of methods are available for sampling bees, including passive trapping and active netting. Much of our knowledge of the effectiveness of these methods has been obtained in nonagricultural environments. A review of bee sampling methodology with a focus on tropical forests and agroecosystems is available [25], as is a frequently updated compendium of practical information on bee biology, collection, processing, identification, and curation [26].

### 2.1. Bee Trapping Methods

**Figure 2:**
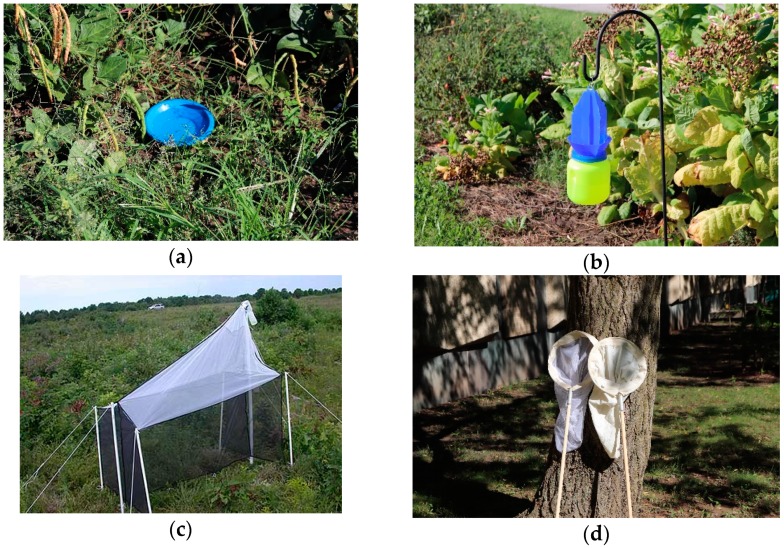
Common sampling methods for non-*Apis* bees: (**a**) Blue bowl trap, Moericke trap, or pan trap; (**b**) Blue vane trap; (**c**) Townes-style Malaise trap; and (**d**) aerial (left) and sweep nets. Attribution: (**c**) Ceuthophilus [60].

The three most commonly used trapping methods for collecting bees for biodiversity studies are bowl traps, vane traps, and Malaise traps. Bowl traps (Figure 2a) are by far the most popular trap type for sampling bees. They are inexpensive, require little collector experience, and large numbers of traps can be deployed and serviced in a time-efficient manner. Bowl traps are sometimes referred to as “Moericke traps”, “bee bowls”, or “pan traps”, although the latter terminology can be confusing because actual aluminum pans are sometimes used in trapping studies [31]. Bowl traps generally consist of a colored plastic bowl or cup containing water or other liquid to serve as a killing agent, with a small amount of fragrance-free dish detergent included to break surface tension. Traps are generally deployed in the morning and samples collected in the late afternoon, or sometimes left out for 24 h, but for longer-term sampling propylene glycol—a better preservative with lower evaporation rates—can be used [32]. Bowl traps are generally placed on the ground, but are sometimes elevated to match the height of most foraging bees. Bowl traps positioned one-third of the way up into the canopy of a Michigan highbush blueberry (*Vaccinium corymbosum* L.) planting collected greater numbers of bees than traps at lower or higher elevations [33].

Small bees such as halictids are often abundant in bowl traps [34,35], but the relative effectiveness of bowl traps in collecting other small bee species is unclear. There is also substantial evidence for taxon-specific bee attraction to certain colors, and a combination of blue, white, and yellow bowls is frequently used to collect a wide diversity of bees [6,36]. Fluorescence may enhance bee captures, and bee collections could also be affected by subtle variations in bowl and color characteristics among manufacturers. Consistent use of bowl traps of standardized design and paint characteristics can address this concern [26].

Traps are generally arranged systematically in a transect or grid with regular spacing to facilitate easy location. Traps placed too closely together may compete for bees; evidence from a large-scale study of bowl trap collections in a variety of ecoregions indicates that bee capture rates plateau at intertrap distances of 3 to 5 m, with no further increase in captures at greater distances [34]. In general, a 30-bowl transect appears to be adequate for assessing bee species richness, with little increase in richness with greater numbers of bowls [37].

Vane traps (Figure 2b) consist of two colored plastic cross vanes (blue or yellow) with a collection container underneath. Insects fall into the collection container after contacting a vane. Vane traps are more expensive than bowl traps, bulkier to transport, and require a support structure for hanging. Vane traps have been used less frequently than bowl traps in bee surveys, and there is a lack of studies on optimal trap number and spacing. There is substantial evidence that vane traps collect relatively large proportions of larger bees such as *Bombus* and eucerine bees [6,7,16,38,39,40], especially long-tongued *Bombus* such as *B*. *auricomus* [16]. It has been suggested that blue vane trap light reflectance may attract these bees by mimicking that of their preferred host plants [16,41]. Large numbers of these large-bodied bees have been collected by blue vane traps in some studies [38,40], suggesting that these traps can collect excessive numbers of these bees if not monitored closely. Because there is a single source of vane traps, standardization is increased by avoiding differences in proprietary pigments of different brands such as may occur with bowl traps [41]. However, long term monitoring projects based on vane traps could be compromised if manufacture of these traps ends.

Bowl traps and vane traps have been used to assess bee abundance, diversity, and species composition in agricultural systems. Blue, white, and yellow bowl traps, as well as blue and yellow vane traps, were compared in Pennsylvania, USA apple orchards [41]. Highest species richness estimates were found for blue vane and blue pan traps, and blue vane traps produced significantly higher species accumulation rates than did all other traps. Vane traps were also deemed less labor intensive to use because they only had to be collected weekly instead of daily, presumably because of low rates of evaporation of preservative compared to bowl traps. Blue vane traps collected a high ratio of common to rare bees compared to the other trap types, resulting in less efficient accumulation of new species per bee collected, and suggesting that oversampling by blue vane traps may be a concern in this system [41]. However, oversampling is of less concern if the goal is to document the common species in a community rather than compiling a comprehensive species list.

Bee communities of Michigan, USA commercial potato fields were sampled using blue vane traps at canopy height and blue, white, and yellow bowl traps elevated 1 m and placed on circular plastic platforms with one of each color trap [40]. Blue vane traps collected 30% more species than did bowl traps. Blue vane traps also collected different species composition, with large-bodied Apidae collected in relatively large numbers compared to bowl traps. However, it should be noted that in this study the bowl traps on each platform were in close proximity to each other; in most studies bowl traps are spaced apart in a transect. The pattern of trap deployment could affect bowl trap bee collections. Blue vane traps may have high relative attractiveness in simple landscapes and collect relatively large numbers of oligolectic bees, perhaps because of the greater relative attractiveness of the UV reflectance of these traps in simple landscapes where resources are scarce [40,42,43]. Blue vane traps were also used in combination with active netting to sample bees in sour cherry orchards in Michigan and Pennsylvania as well as apple orchards in Pennsylvania [16]. The blue vane traps collected an assemblage of bees distinct from that collected via active netting, and the trap collections included primarily species that were not collected from orchard crop flowers. Several studies in a variety of habitats have shown blue vane traps to be superior to yellow vane traps in collecting bee abundance and species richness [6,7,41,44]. In locations where oversampling by blue vane traps is a concern, the lower effectiveness of yellow vane traps could be an advantage, and replacement of some blue vane traps with yellow vane traps or perhaps bowl traps might reduce excessive sample sizes. Use of a combination of trap types and colors could also increase the proportion of the bee species richness collected (see below).

While vane traps have been used to assess bee abundance and diversity in a variety of environments, bowl traps are more commonly used and appear to be the method of choice for monitoring bees at large geographic scales [45]. A protocol for monitoring insect pollinators and detecting declines at such geographic scales using bowl traps has been proposed [46,47] and the merits of this proposed program debated [48,49].

Bowl traps and vane traps are the most commonly used trapping methods in bee monitoring and surveillance, but other trap types are available as well. Malaise traps (Figure 2c) are large mesh fabric traps that are often used in collecting bees and other flying insects, which tend to move upwards into the collection container upon contact with the trap [50]. There are several Malaise trap designs, but the most widely used is the Townes-style Malaise trap with a black base and white roof [51,52]. Malaise traps have frequently been used to sample various insect groups in a variety of environments [6,53,54,55,56,57]. Only one of these studies focused on bees in an agricultural setting. This study investigated bee diversity of southern Costa Rican coffee landscapes at 11 sites, including shaded coffee farms, unshaded coffee farms, and nonagricultural sites, using Malaise traps [56]. Unshaded coffee farms had higher bee abundance and species richness than the other two environments, and Malaise traps collected substantial numbers of small-bodied bees, many of which had never been reported as common in coffee farms. Although done in a very different environment, studies in Illinois, USA restored tallgrass prairie [6] and deciduous forest [7] suggest that the results of the Costa Rica study may be at least partially related to better relative performance of Malaise traps in open vs. shaded environments. In the Illinois studies, Malaise traps collected by far the greatest bee abundance and species richness of the four methods used in restored tallgrass prairie, but collected very few bees in nearby deciduous forest, perhaps because of reduced phototactic behavior of bees in a closed canopy environment. A significant association between Malaise trap collections and small-bodied bees has also been found [58], supporting the observation that these traps are efficient in collecting small bees. This could be advantageous in facilitating sampling of smaller, lesser known bees that are infrequently collected in agroecosystems.

Because Malaise traps are probably more “passive” than bowl traps and vane traps, which incorporate color to attract bees, it is possible that Malaise traps collect a more representative sample of the overall bee fauna at a particular location, although color and fabric mesh size have been shown to influence collections of some insect groups such as tabanids [59] and very small hymenopterans [54]. However, Malaise traps also collect many nontarget insects, which is a drawback unless this bycatch is put to good scientific use. While large numbers of bees can be collected with Malaise traps and sample collection time is low once traps are set up, the typically abundant bycatch means that time spent sorting out the bees is relatively great.

Other disadvantages of Malaise traps are cost (200+ U.S. dollars per trap) and significant set-up time, limiting the number of traps that can be deployed. This can be a problem because there is evidence that Malaise trap effective trapping area is localized, with large variation among traps, at least in tallgrass prairie where floral resources and bees have a patchy distribution [57]. This problem is compounded because Malaise traps are also time-consuming to relocate after set up. However, in many agroecosystems bee distributions may be more uniform with less variation in bee collections among traps, meaning fewer traps would be required. Studies of spatial variation in Malaise trap collections of bees are needed in these environments. Smaller, more portable Malaise traps such as “SLAM” traps (sea, land, and air Malaise traps) may provide an acceptable alternative to traditional Malaise traps; research on the effectiveness of SLAM traps for collection of bees is needed as well.

Comparisons of Malaise traps with bowl and vane traps have not been done in agroecosystems, but in Illinois, USA restored tallgrass prairie and deciduous forest these methods collected somewhat distinct bee species composition, and certain species (indicator species) were significantly associated with particular trap types [6,7]. Furthermore, these trap types varied in the functional traits of bees collected in this tallgrass prairie [58]. Ground-level pan traps collected statistically greater numbers of solitary vs. social and above- vs. below-ground nesting bees than expected. Malaise traps collected greater numbers of polylectic vs. oligolectic bees as well as bees of shorter body length, and vane traps collected greater numbers of longer body length bees than expected. Comparisons of the performance of these traps in agroecosystems, from both a species and trait diversity standpoint, could yield important information not only on variation in trap performance but also on the ecology and natural history of bees in environments of varying complexity.

### 2.2. Active Netting of Bees

In addition to trapping, bees are also commonly sampled using insect nets. Insect netting generally takes two forms: targeted netting of bees using an aerial net (Figure 2d, left), and sweeping of vegetation using a sweep net (Figure 2d, right). Targeted netting allows the researcher to preferentially collect particular species, and to associate an individual bee with the particular flower it visits. Aerial nets are generally constructed of a lightweight mesh fabric, whereas sweep nets are constructed of sturdy cloth that allows the collector to sweep the net through vegetation, collecting insects that are on the vegetation [61]. While less focused than targeted netting, sweep netting can provide information on the bee fauna associated with a particular area of vegetation. In both types of netting, samples can be standardized by timing the sampling period or by measuring the distance or area covered. Active netting of bees is inexpensive, but relatively labor-intensive, and variation in collector abilities can make comparisons among samples difficult. Targeted netting, if done thoroughly enough, can provide a relatively complete sample of the bee fauna present, or can supplement bee samples collected by other methods. For instance, relative to bowl traps, targeted netting can yield high numbers of fast or high-flying bees such as *Colletes*, *Megachile*, and *Melissodes* [62]. However, depending on collector experience and skills, smaller and more cryptic bees may be under-represented in targeted netting samples.

Targeted netting in combination with bait stations is a well-known method of sampling orchid bees (Apidae: Euglossini), a dominant bee taxon of Neotropical forests that includes pollinators of economically important crops such as rubber trees, *Hevea brasiliensis* Müll. Arg., and Brazil nuts, *Bertholletia excelsa* Humb. & Bonpl. (Figure 1d) [30,63,64,65]. Male orchid bees visit orchids and other flowers to collect aromatic chemicals that appear to be important in species recognition and mate choice [66]. These compounds make it possible to sample orchid bees by netting them as they approach a bait station. Orchid bee bait stations generally consist of an absorbent material such as cotton suspended from a string and baited with a chemical attractant. Generally, several bait stations are set up a few meters apart, with each station being baited with a different attractant. There are dozens of these chemical attractants [66]; some are relatively expensive, and may not be available in some countries, necessitating international shipping which adds to the expense. Some of the more commonly used attractants include benzyl acetate, cineole, eugenol, methyl salicylate, and vanillin. Orchid bee attractants vary greatly in the abundance and species composition of bees attracted [66,67]. These attractants are also used in conjunction with traps to collect orchid bees. However there is evidence that baited traps collect lower abundance and species richness than does sampling with baits and active netting, with trap collections biased toward larger orchid bee species in the genera *Eulaema* and *Eufriesea* [68]. A review of these and other methodological issues related to orchid bee sampling is available [69].

Sweep netting generally involves continuously sweeping vegetation with the net for a specific time period, while walking a measured transect, or covering a specified area. This allows the collector to capture relatively small or inconspicuous bees that may not be collected readily by targeted netting. Sweep netting is a commonly used method of sampling bees in tropical locations [25], but appears to be less frequently used in temperate regions. In temperate agroecosystems, sweep netting has been used in conjunction with bowl traps to assess non-*Apis* bee abundance and diversity in canola fields in Alberta, Canada [70,71,72], grasslands of agriculturally dominated landscapes in Iowa, USA [73], and lowbush blueberry fields in Maine, USA [74]. In the studies of bees associated with canola fields, over 20 times as many bees were collected in bowl traps as by sweep netting [72], but difficulty in assessing relative sampling effort makes comparisons between methods problematic.

Approximately 5% of all bee and wasp species are “trap-nesting” species, that is, they establish nest sites in tunnels in dead wood or grass stems [75]. These include species in the genera *Megachile*, *Osmia*, *Hylaeus*, *Trypoxylon*, and *Ancistrocerus*, among others. These species can be sampled using “trap-nests” constructed of wood or other materials. Trap-nests have been used in a variety of ecological and life history studies, including a recent study of the phenology of plant flowering and solitary bee and wasp emergence in Rocky Mountain, USA meadows [76]. Trap-nests appear to be an uncommon approach in agroecosystems, but have been used to assess nesting frequency of trap-nesting species in fallow and wildflower plots in agriculturally dominated areas in North-Central Florida, USA [77]. Trap-nests were also used to examine effects of landscape complexity on trap-nesting Hymenoptera and their parasites in a region dominated by intensively managed agriculture in northwestern Germany [78]. In addition to detection of trap-nesting species, interesting life history data, such as seasonality, duration of life stages, and nest parasites can be collected using this method. The construction of trap-nests can be labor intensive, although premade “bee houses” are commercially available and could be useful for research. Mold formation can be a problem in trap-nests, especially if an impervious material such as glass tubing is used. Naturally occurring plant materials such as bamboo are good for constructing trap-nests, but the variable diameter is a disadvantage. Straight-grained pieces of seasoned white pine have been found to work well for constructing trap-nests [75]. Further studies on the usefulness of this method in sampling beneficial Hymenoptera in agroecosystems are needed.

## 3. Natural Enemies/Pest Management

**Figure 3:**
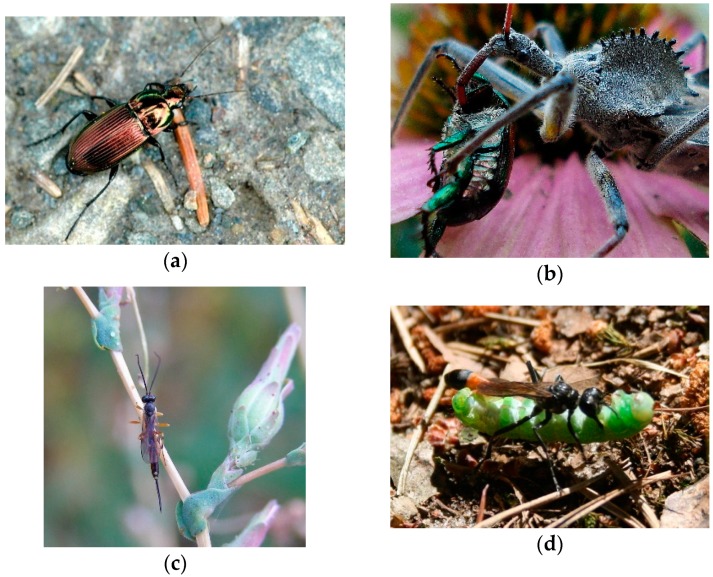
Some natural enemies potentially important in insect pest control: (**a**) a carabid beetle, *Poecilus cupreus* (L.); (**b**) the predatory wheel bug, *Arilus cristatus* (L.) consuming a Japanese beetle, *Popillia japonica* Newman; (**c**) a campoplegine ichneumonid; (**d**) the hunting wasp *Ammophila sabulosa* (L.) carrying a caterpillar. Attributions: (**a**) James Lindsey at Ecology of Commanster [79]; (**b**) Andy McLemore, [80]; (**c**) H. Dumas, [81]; and (**d**) Patrick Reijnders, [82].

The value of natural control of native agricultural pests by beneficial insects (Figure 3) has been estimated at $4.49 billion [1]. However, agroecosystems often provide an unfavorable environment for the survival and reproduction of these beneficial organisms. Conservation biological control, the manipulation of the environment to conserve these beneficial natural enemies and enhance their effectiveness, is an increasingly important component of agricultural pest management [5]. In order to evaluate the status of these natural enemies and the effectiveness of conservation measures, reliable sampling methods are needed. In this section I review methodological approaches to sampling and monitoring these beneficial arthropods in agroecosystems.

### 3.1. Sampling Natural Enemies in Epigeic Environments

#### 3.1.1. Pitfall Traps

**Figure 4:**
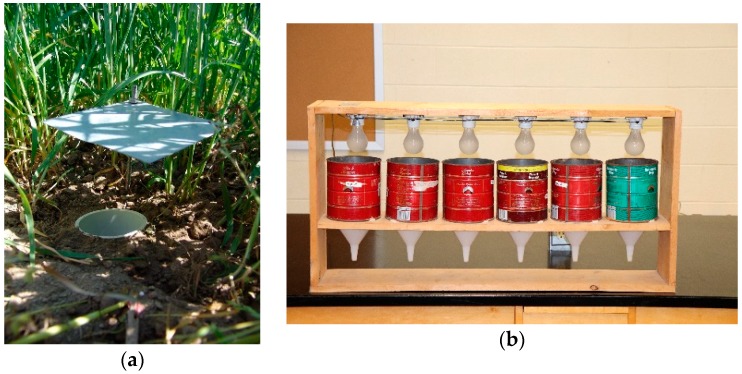
Two common sampling methods for epigeic arthropods: (**a**) Pitfall trap or Barber trap and (**b**) homemade Berlese–Tullgren funnels. Attribution: (a) Mnolf [110].

The most important groups of beneficial epigeic natural enemies include carabid beetles, (Coleoptera: Carabidae) (Figure 3a), rove beetles (Coleoptera: Staphylinidae), wolf spiders (Araneae: Lycosidae) and other wandering spiders, and centipedes (Myriopoda: Chilopoda). Of these groups, carabids are the most thoroughly studied and well understood. Carabids and other epigeic arthropods are most commonly sampled using pitfall traps (Figure 4a) [83,84,85]. This trap type is the method of choice for carabid abundance and diversity sampling within the National Ecological Observatory Network (NEON), a continental-scale program to increase our knowledge of the ecological impacts of climate change, land-use patterns, and invasive species on biodiversity and ecosystem processes in the USA [86]. Pitfall traps have been used to collect and quantify epigeic arthropods since at least the early 1900s [87,88]. Pitfall traps consist of an open-top container buried in the ground with the rim flush with or slightly below the substrate surface. Animals falling into the container are trapped. There are many modifications of the basic pitfall trap design, including a funnel to prevent escape, a cover to block rain and debris from falling into the trap, baits to target particular taxa, and drift fences and barriers to increase captures and infer direction of movement [89,90]. Pitfall traps are sometimes used dry to collect live specimens, but predatory species feeding on each other is often a problem [91], so more often the trap is partially filled with a preservative to kill and maintain the trapped arthropods. A variety of preservatives have been used, including water, brine, formalin (which is highly toxic and generally no longer used), ethylene glycol, propylene glycol, acetic acid, alcohol, kerosene, and chloral hydrate [85]. Because the numbers of specimens captured is dependent on their activity levels as well as their abundance [92], pitfall trap samples are best described in terms of “activity-abundance” or “activity-density” [84].

Most studies using pitfall traps in agroecosystems have focused on carabids. Many of these have been done in the agriculturally important Midwestern USA [93]. One focus has been on effects of cropping practices on beneficial arthropods. For example, pitfall traps and quadrat sampling have been used to examine the effects of organic transition systems on carabids and other arthropods in Illinois, USA agricultural fields [94,95]. Pitfall trapping indicated that activity levels of beneficial arthropods were reduced in the low intensity system, but quadrat sampling showed that this environment had the greatest density of these arthropods, suggesting that the more stable environment of the low intensity system resulted in less arthropod movement. Predation rates on sentinel wax moth (*Galleria mellonella* L.) larvae and seed removal were greatest in the low intensity system as well, suggesting that low intensity agricultural practices were advantageous to beneficial arthropods. These results demonstrate that a combination of sampling methods can often provide more information and greater insight than does a single method. Pitfall traps have also been used to monitor carabid abundances in conventional vs. reduced-risk insecticide management practices in Michigan, USA highbush blueberry plantations [96], to assess potential effects of transgenic corn on carabid abundance in Iowa, USA [97], and to evaluate the potential of carabids to serve as indicators of adverse environmental effects of genetically modified crops in Europe [98].

In addition to assessments of beneficial arthropods in relation to conditions within the crop environment, there have been several landscape-scale investigations of effects of anthropogenic land use patterns on arthropods, based on pitfall trap sampling. Pitfall traps were used to show that carabid community variation is associated with extent of disturbance in sites ranging from highly disturbed pasture to cocoa (*Theobroma cacao* L.) plantations to successional forest and undisturbed native forest in the Brazilian Amazon [99]. This study also identified several carabid indicator species and suggested that carabid communities can be used to evaluate the status of Neotropical ecosystems. Pitfall traps were also employed to compare responses of epigeic spiders and carabids to differences in cultivated and semi-natural environments in China [100]. Results of this study indicated that the high plant diversity and vegetation structure of semi-natural habitats significantly enhanced spider abundance, whereas these factors were a poor predictor of carabid abundance and species richness. Plant diversity affected the species composition of both groups. These studies illustrate the utility of pitfall traps for investigating associations between habitat characteristics and beneficial epigeic arthropods. However, the potential for habitat-specific effects on collections of arthropods in pitfall traps should be considered, and if possible a combination of sampling methods should be used.

Like most trapping methods, pitfall traps are subject to certain biases, and collections can be affected by a variety of factors. In a study of the spider community of winter wheat, species composition and sex ratios obtained using pitfall traps differed from that obtained via density sampling using a D-Vac suction sampler (see next section). These discrepancies could be due to species-specific variation in activity patterns, searching for mates or food, and species-specific differences in ability to escape from the pitfall traps [101]. Challenges associated with pitfall trapping have been studied in detail for carabids [9,102]. As previously mentioned, arthropod activity level affects the probability of capture. This may be related to another bias associated with pitfall traps—the tendency for over-representation of large-bodied species, as shown by a study in which carabid composition of pitfall trap samples was compared with that collected via extraction from litter using Berlese–Tullgren funnels (Figure 4b) [9]. This is probably related to locomotive abilities. Larger species generally move greater distances than smaller species, whereas smaller species such as *Notiophilus* spp., often evade capture upon reaching the lip of the trap [9,102,103]. Different carabid species also have differing abilities to escape from traps [104]. Pitfall trap collections also vary in relation to diel activity patterns of carabids, with diurnal carabids being under-represented in pitfall traps, perhaps because they can see and avoid the traps [102,103]. However, diel activity of particular carabid species can vary in relation to habitat, with greater or lesser diurnal activity in agricultural fields than in nearby uncultivated habitat [105]. Amount and structure of vegetation can also affect carabid collections. For instance, pitfall traps with vegetation removed from the surrounding area generally collect more carabids than do traps without a cleared area, although different carabid species vary in their response to different vegetation structure [102]. Characteristics related to the pitfall traps themselves can also affect carabid collections. Round traps without covers tend to collect greater abundance of carabids than do covered traps or rectangular traps, although there is some species and habitat-related variation [9]. Type of preservative can influence abundance and species composition of collections as well [106,107], so consistency in comparative studies using pitfall traps is important. On the other hand, variation in pitfall trap collections related to trap variation can be put to good advantage in species inventory studies because the traps may collect greater combined species richness [106]. A review of factors that may affect arthropod collections by pitfall traps is available [106].

There is evidence that pitfall trap samples can be related to densities of carabids [108]. Fenced pitfall traps were evaluated for use in estimating densities of epigeic predatory beetles, using mark-release-recapture. For seven of 10 species studied recapture rates were greater than 70%, suggesting that these traps are suitable for estimating densities if trap density is sufficient [109]. Further studies are needed to evaluate the effectiveness of pitfall traps in agroecosystems and how sampling is affected by arthropod behavior, activity levels, and environmental variables such as vegetation structure. Studies incorporating a second sampling method that measures arthropod densities, such as quadrat sampling and extraction of arthropods [9,95], would allow comparison of pitfall trap samples with density estimates of epigeic arthropods. Some of these approaches to measuring epigeic arthropod densities are discussed in the next section.

#### 3.1.2. Other Methods of Sampling Epigeic Arthropods

While pitfall traps are by far the most commonly used method of sampling epigeic arthropods, other methods are available as well, some of which can provide estimates of arthropod density. Other methods for sampling epigeic arthropods include suction samplers, emergence traps, soil flooding, microhabitat removal and arthropod extraction, and cryptozoan boards.

Suction samplers are frequently used to collect arthropods from vegetation, but can also be used to collect epigeic arthropods. These samplers use suction provided by a motorized fan to collect arthropods into a collection net. The most widely used suction sampler is the Dietrick vacuum insect net, or “D-vac” [111,112]. When used to sample a specified surface area, they can provide a measure of arthropod density [101], but sampling within enclosures that are sealed to the ground is needed to avoid collecting specimens from outside the measured sampling area [113]. In terms of sampling of beneficial arthropods, cursorial spiders have been sampled in Central European farmland and perennial habitats using suction sampling [114]. Sampling efficiency may be taxon-specific, and large, heavy predators are often undersampled [115]. Wet sampling locations, such as when dew is present, can reduce collections because specimens stick to the nozzle, hose, and collection net [116].

Emergence traps generally consist of a cloth or metal enclosure that covers a known area of soil/litter or other environment. When the animal exits as it moves from one part of its habitat to another, perhaps during a specific stage of its life, the animal is trapped in a collection container [115]. Emergence traps are also known as eclectors, or photoeclectors if the trap exploits positive phototaxis of emerging organisms [112], as most emergence traps do. Emergence traps can provide a measure of density since they collect from a discrete area, and are also advantageous in that specimens collected are from the location of interest rather than potential migrants. Typically an emergence trap will include a pitfall trap within it to collect invertebrates that tend to stay on the ground rather than moving up to the collection container, and in this discussion “emergence trap” is considered to contain a pitfall trap as part of the apparatus.

Emergence traps have been used in agricultural studies, although less frequently than pitfall traps. Emergence traps, along with pitfall traps (separate from the emergence traps), were used in a study that showed oilseed rape to be a beneficial crop environment for at least two dominant species of carabids, *Poecilus cupreus* (L.) and *Brachinus sclopeta* (Fabricius), in western France [117]. The two sampling methods allowed the authors to investigate spring activity-density as well as overwintering location of these carabids. Emergence traps and pitfall traps were also used to assess the effects of agricultural land use on carabid community trait composition in southwestern Sweden [118]. Emerging carabids in high intensity land-use fields had lower body length, whereas crop fields produced greater proportions of carabids with good flight ability and carnivorous diets than did grasslands. Species composition differed significantly between sampling methods, and there was a significant land-use type by sampling method interaction. Macro-emergence traps (modified Malaise traps) have been used successfully to sample arthropod natural enemy communities overwintering in semi-natural habitats in agricultural landscapes of southwestern France [119]. A variety of arthropod taxa were collected, including Araneae, Hemiptera, Neuroptera, Carabidae, Coccinellidae, Syrphidae, Staphylinidae, and various parasitoids.

Emergence traps appear to have a great deal of potential for studies of agroecosystem invertebrates. They are somewhat complimentary with pitfall traps in that they provide direct density estimates of an enclosed area whereas pitfall trap collections to a great extent reflect activity levels of the organisms. Therefore, use of these two trap types in the same habitat, and comparison of the invertebrate assemblages collected with the two methods, can provide important information on the biology of these animals [112]. One potential drawback of emergence traps is that they can influence the microclimate of the enclosed area and so may influence the phenology or behavior of the invertebrates. These effects can be specific to trap construction [115]. For instance, metal-screened emergence traps collected twice as many pink bollworms, *Pectinophora gossypiella* (Saunders) as did plastic-screened ones [120].

Soil flooding involves saturating a defined area of soil with water, which forces the arthropods to the surface where they can be collected. This method was originally used to collect carabids and staphylinids in riparian habitats [121] and provides an estimate of density. An area of soil is isolated with a 0.1 m^2^ enclosure, vegetation is examined and removed, and 2 L of water is poured into the enclosure. Arthropods are removed, and later an additional 2 liters are added and arthropods are again removed [112]. Studies in a variety of crop environments have shown that small carabids, staphylinids, and Araneae can be collected via soil flooding [122,123,124]. In a study using three methods to sample beneficial arthropods of agricultural fields in Austria, small carabids were predominant in soil flooding and emergence trap samples, whereas the larger *P*. *cupreus* was predominant in pitfall traps, probably reflecting its greater mobility [124]. However, soil flooding can be ineffective under certain soil conditions. Blockage of the soil surface by silty mud can inhibit the emergence of arthropods, and in dry clay soils with large cracks or other highly perforated soils, impractical amounts of water can be required to sufficiently flood the soil [112].

Microhabitat removal and arthropod extraction involves removal of a known area of litter or soil for later extraction of arthropods, providing an estimate of density. A variety of methods have been used. Samples can be hand-sorted and searched for arthropods manually, or can be placed in an apparatus for extraction [112]. The most widely used of these is the Berlese–Tullgren funnel (Figure 4b), in which the sample is placed in a container with a mesh screen to support the sample and a funnel that leads to a collection container below. A light bulb or other heat source is located above the sample; the arthropods within the sample are forced downward into the collection container by the heat and desiccating soil [115]. Many modifications and improvements have been made on the original design. When placed in the extractor, the soil core should be inverted and kept intact; otherwise, the natural passageways will be disrupted and the arthropods’ “escape routes” will be eliminated [125]. It has also been noted that significant losses can occur through arthropods becoming trapped in the condensation that forms on the sides of the container from the moisture contained in the soil core; space should be left between the core and the sides of the container to prevent this [126].

Berlese–Tullgren funnels have been used to collect spiders from samples taken from maize, ryegrass, and pasture [127], and beneficial predatory arthropods (spiders, carabids, and staphylinids) from samples taken from overwintering sites within cereal fields [128]. However, the effectiveness of this method can vary with habitat. Berlese–Tullgren funnels were found to be an ineffective method for sampling epigeic arthropods in the sandy soils of Florida, USA bush bean plots, because of the low amount of surface litter and large amounts of sand that fall from the sample into the collection jar [129].

Cryptozoan boards, sometimes referred to as “drop boards”, can be used to collect cryptozoans, the “hidden animals” that shelter and hide underneath stones and logs [115,130]. These are generally wooden boards of 0.1 m^2^ each that are placed on the ground. When collecting samples, the boundaries of the board should be marked to avoid sampling outside of the covered area. The board should be lifted and specimens collected quickly, since many of these animals move rapidly and quickly find hiding places. An aspirator for sucking up the specimens is helpful [130]. A potentially useful alternate method is to place a 0.1 m^2^ enclosure around the board (slightly greater area than the board), with the edges pressed into the soil. Then the board is removed and specimens collected, with the enclosure preventing escape. The specimens can be collected on site, or the litter and soil can be collected and placed in a bag for later sorting in the lab, or perhaps extraction using Berlese–Tullgren funnels. Another possibility might be to use a suction sampler (see above) to collect the material under the board, although the high humidity of the microhabitat might cause clumping of the litter and so preliminary breaking up and loosening of the material may be necessary.

Predatory macroarthropods found among the cryptozoa include carabids, centipedes, staphylinids, dermapterans, and Araneae. The microhabitats these animals are adapted to are generally dark, with fairly stable temperature and humidity compared to the surrounding environment [131]. These animals vary in the degree to which they are bound to this environment, and predators appear to be less restricted to the cryptozoan microhabitat than other organisms [132]. Because it may take some time for the microenvironment underneath a cryptozoan board to develop into an optimal one for cryptozoan colonization, the length of time the cryptozoan boards are left in place may affect collections, and this may vary in a taxon-specific manner. Abundance and taxonomic composition of invertebrates collected via cryptozoan boards can also vary with board size, color, and surface features of the ground [133].

Cryptozoan boards have been used relatively infrequently in agroecosystems. In Zimbabwe, millipedes are important in the processes of decomposition and nutrient cycling [134]; the seasonal activity patterns of these animals has been examined in agricultural pasture and other habitats using cryptozoan boards [135]. Cryptozoan boards were also used to sample soil surface arthropods in the aforementioned study in Florida, USA bush bean plots. Cryptozoan boards collected less taxonomic diversity than did pitfall traps but more than Berlese–Tullgren funnels overall. Compared to pitfall traps, cryptozoan boards collected fewer staphylinids, spiders, parasitoid wasps, and various other taxa; more crickets, Dermaptera, and elaterids; and similar numbers of formicids and carabids [129]. Cryptozoan boards are generally less productive than pitfall traps on a per unit time or effort basis [136].

### 3.2. Sampling Natural Enemies on Vegetation

#### 3.2.1. Direct Counts

In some cases, beneficial arthropods can be sampled using direct counts of individuals on vegetation. This method is best used on relatively inactive arthropods; accurate counts are difficult to obtain for active, fast-moving, or easily disturbed arthropods. In fields with regularly spaced crops, natural enemies associated with the crop may be visually counted and densities per plant or per unit area calculated. But in more structurally complex agroecosystems, a measure of density can be obtained using quadrats or specific plant structures as the sampling unit. Spiders of grassland and cereal crop vegetation have been sampled using direct counts within 1 m^2^ quadrats [137]. Eggs and larvae of syrphid and coccinellid predators of aphids were counted on winter wheat shoots and in quadrats. The latter study combined those two data sets to obtain predator density estimates for the entire habitat [138]. Some predators may have highly clumped distributions related to prey density and other environmental factors. Coccinellids, for instance, are affected by temperature and aphid density. When these factors were included into a predictive model of coccinellid densities in wheat fields, R^2^ values of 0.63 to 0.94, depending on species, were found between coccinellid visual counts and population densities [139]. Direct visual counting of arthropods is relatively labor-intensive, and this can be a major disadvantage, depending on the difficulty of seeing and counting the arthropods in question and the complexity of the environment in which they live. Sixty to 90 person-minutes per plot were required to search for and remove spiders from 0.5 m^2^ plots of grassland with a mean vegetation height of 15 cm [140].

More active arthropods can be counted after removal from vegetation using a suction sampler, described previously for epigeic arthropods. This method has been used to quantitatively sample the spider fauna of USA Great Plains winter wheat within enclosed 1 m^2^ sample plots [141].

#### 3.2.2. Sweep Netting

Sweep-netting (Figure 2d, right) is a commonly used method of sampling arthropods on vegetation [115], and can collect a variety of beneficial arthropods including spiders as well as predatory hemipterans (Figure 3b), beetles, and neuropterans. Sweep-netting was used in the above study estimating coccinellid densities in wheat fields [139]. Regression models relating sweep net collections to absolute densities of coccinellids produced R^2^ values ranging from 0.51 to 0.90, depending on species. Effects of different crop types and production systems on densities of staphylinids have been examined using sweep-netting and four other methods in Punjab Province, Pakistan, but comparisons among sampling methods were not done [142].

Sweep-netting has important advantages, including low equipment cost and potentially large yield of specimens per unit effort. By standardizing efforts based on number of sweeps, area sampled, or duration of sampling, repeatable and comparative results can be obtained. A formula allowing reporting of sweep-netting samples as insects collected per unit volume of vegetation has been developed [143] and applied to coccinellid sampling [144].

If sweep net samples obtained by different people are included in a comparative study, then efforts should be undertaken to ensure that the samples are standardized and comparable. There are other potential biases and sources of error associated with sweep-netting. Arthropods that tend to fall off the vegetation or fly away as the netter approaches will be undersampled [130]. Vegetation structure and the taxon being sampled can also affect sweep net collections. If the species being sampled is distributed throughout the vertical range of the plants, then smaller proportions of the species will likely be sampled from taller plants relative to shorter plants [115], and even closely related species can differ in their susceptibility to collection by sweep net [145].

#### 3.2.3. Suction Samplers

As mentioned previously in the section on sampling epigeic arthropods, these devices use suction provided by a motor to collect arthropods. They are frequently used to sample arthropods from vegetation. These devices have been used extensively in sampling of grassland invertebrates, but are also useful in agroecosystems, including alfalfa [146], cereals [147,148], and winter wheat [149]. Efficiency of vacuum samplers is taxon-specific, can vary with year, season, site, and vegetation structure, and can be affected by a variety of environmental factors [147,148,150,151]. High abundance and diversity of spiders (4700 individuals and 91 species) were sampled in Central European farmland and perennial habitats using suction sampling [114]. Per unit effort, suction sampling collected greater abundance and species richness of arthropods than did sweep-netting in California coastal sage scrub habitat [152].

#### 3.2.4. Shaking and Beating Vegetation

Arthropods can be sampled from vegetation by shaking or using a stick to beat plants or plant structures, and collecting the arthropods that fall from the vegetation in a collection tray or cloth [115]. The insects can be rapidly collected with an aspirator, or into a tray containing a killing solution/preservative such as ethyl alcohol. Large arthropods that are easily dislodged, such as many pentatomids, may yield absolute estimates, but of course insects that tend to fly away when disturbed will be poorly sampled [115,130,153]. Predatory arthropods have been successfully sampled from apple trees by removing branches over a cloth, and then cutting into smaller sections and putting these into a revolving rectangular shaker and dislodging the insects [154]. Beating has been used to sample coccinellids on trees and shrubs in a study of the impacts of the invasive coccinellid *Harmonia axyridis* (Pallas) on native coccinellids [155]. Shaking or beating may substitute for sweep netting when shrubs and small trees are being sampled, or in dense, thorny habitats that are not suitable for sweeping. It has been pointed out that beating could damage some plants (and therefore disrupt the habitat being sampled), and for this reason shaking may be preferable [156].

### 3.3. Sampling Predatory and Parasitic Wasps and Flies

#### 3.3.1. Trapping Methods

Since many predatory and parasitic wasps and flies (Figure 3c,d) are active fliers and often abundant on vegetation as they search for hosts, prey, or plant-based food sources, some of the previously mentioned methods, such as netting and suction sampling, can be useful for these insects as well. Trapping methods for these insects include pan traps (Figure 2a) (also called Moericke traps, or, in the bee literature, bowl traps), Malaise traps (Figure 2c), and sticky traps. These traps have been used in studies of parasitoids in in a variety of habitats including agroecosystems and other cultivated habitats. For instance, *Liotryphon crassiseta* (Thomson), an ichneumonid parasitoid of the red-belted clearwing moth, *Synanthedon myopaeformis* (Borkhausen), was monitored using yellow pan traps in apple orchards of western Poland [157]. Malaise traps were used to compare relative abundance of parasitoids in herbicide-treated and untreated loblolly pine (*Pinus taeda* L.) plantations in the coastal plain of Georgia, USA [158]. The effectiveness of different sticky trap colors was compared in a study of aphelinid parasitoids of San Jose scale in apple orchards in North Carolina, USA. Trap color had a significant effect on relative abundances of species collected. Among colored traps, traps combined with San Jose scale sex pheromone collected significantly more *Encarsia perniciosi* (Tower) than did unbaited traps, suggesting that this pheromone acts as a kairomone for this parasitoid. Black baited traps collected significantly more *E*. *perniciosi* than did baited yellow or white traps, suggesting an olfactory–visual interaction in host location by this species [159]. Yellow sticky traps have also been used to monitor abundances of *Cardiaspina* psillids and adult parasitoids during an outbreak in *Eucalyptus* woodland in Western Sydney, Australia [160].

Several studies have compared different methods of sampling parasitic hymenoptera in a variety of habitats. Two Malaise traps and four pan traps (color not given) were used to collect parasitic Hymenoptera in a transect in organic rice fields and a nearby protected area in the Brazilian Atlantic Forest biome [161]. Malaise traps collected the greatest numbers of parasitoids (58% of total), and collected 1.7 to 1.9 times the estimated species richness as did the pan traps. In rice ecosystems of Tamil Nadu, India, sweep-netting, yellow pan traps at ground level, and yellow pan traps elevated to the rice canopy were compared in effectiveness of sampling chalcicid and pteromalid parasitoids [162]. Sweep-netting and ground-level yellow pan traps collected the most parasitoids overall and the most pteromalids, whereas sweep-netting collected the most chalcidids. Elevated yellow pan traps were clearly the least effective method overall, but comparison of pan trap and sweep-netting collection effort is difficult. Sweep-netting, yellow pan trap, and Malaise trap collections of parasitic Hymenoptera were compared at southern India urban sites disturbed by human activities and cattle grazing. Among platygastrids, which were identified to genus level, Malaise traps had the highest capture rate overall, but results varied among genera [163]. For all parasitic Hymenoptera collected, each trap was effective for particular taxa, and it was concluded that a combination of these trap types would be best for comprehensive collections [164]. Malaise traps, yellow pan traps, and flight intercept traps were also compared in secondary tropical deciduous forest on the Caribbean island of Dominica [165]. Yellow pan traps collected the greatest numbers, but relatively low diversity because of the dominance of a few species of diapriids. Malaise traps collected the greatest richness (based on morphospecies) and diversity, while flight intercept traps collected relatively low abundance and richness. Again, comparison of collection effort among different methods is problematic.

Aculeate wasps are also potentially important biological control agents in agroecosystems [166,167,168], and several of the above trapping methods have been used to sample these wasps in a variety of habitats. Malaise traps and sweep nets have been used to collect the crabronid *Bembecinus quinquespinosus* (Say) in a study of male mating and mate location [169], and Malaise traps, sweep nets, and pan traps were used in faunistic surveys of sphecid and crabronid wasps in Iran [170,171].

The Diptera includes many potentially important natural enemies of agricultural pests. Tachinids (Diptera: Tachinidae) have shown potential for biological control of some agroecosystem pests [172,173,174,175], and can be collected using a variety of methods. Tachinid specimens in the diverse subfamily Exoristinae have been recorded as collected in North Korea using sweep net and yellow pan traps [176]. Tachinids comprised 23% of overall parasitoid captures in Malaise traps in Georgia, USA coastal plain loblolly pine plantations, and the tachinid *Lixophaga mediocris* was the most abundant parasitoid of the loblolly pine pest Nantucket pine tip moth, *Rhyacionia frustrana* (Scudder), collected by this method (72% of total) [158]. Many species of syrphid flies (Diptera: Syrphidae) are also beneficial, the larvae as natural enemies and adults as pollinators. Syrphids were studied as landscape indicators in agroecosystems in Italy using Malaise traps and yellow sticky traps [177]. Malaise traps collected the greatest numbers, but some species were collected only by sticky traps, some of the latter in large numbers. Robber flies (Diptera: Asilidae) are voracious predators that can be sampled via targeted netting and Malaise traps [178,179,180]. In Illinois, USA restored tallgrass prairie and adjacent deciduous forest, Malaise traps collected 26 species and 0.35 robber flies/trap/day; approximately 85% of estimated minimum asymptotic species richness was collected [178,179]. Studies on sampling and impacts of predatory flies and aculeate wasps in agroecosystems are needed.

#### 3.3.2. Assessing Parasitism and Predation

Unlike most predation events in which little or no sign of the prey remains, infection by parasitoids can often be detected and estimates of parasitism rates derived. Immature stages of many pest insects can be collected and placed in rearing chambers; the relative proportions of parasitoid and pest adults that emerge can be used to calculate an estimate of parasitism rates [158,181,182,183], sometimes referred to as “apparent parasitism” [184]. Parasitism rates may also be estimated by dissection of the hosts [185,186]. The first method (rearing) may be complicated by failure of some parasites and hosts to emerge. This may necessitate dissection of these individuals, particularly if these emergence failures could potentially affect the parasitism estimate (either parasitoid or host relatively more likely to successfully emerge and be counted, or different species of parasitoids vary in emergence success). Parasitism rate estimates can also be complicated by life histories of some parasitoid taxa. Hyperparasitism can cause the impacts of primary parasitoids to be underestimated, whereas multiple emerges of parasitoids from a single host (associated with gregarious parasitoids or polyembryony) can lead to overestimation of parasitism rates if not taken into account. Identification of parasitoids based on dissections may be difficult depending on the condition or the life stage of the parasitoid.

Parasitism by *Trichogramma* egg parasitoids can be detected based on the blackening of the vitelline membrane of the parasitized egg [187], and this marker has been used to estimate parasitism rates of a variety of lepidopteran pests, including the Nantucket pine tip moth, *R*. *frustrana* [188], tomato leaf miner, *Tuta absoluta* (Meyrick) [189], imported cabbageworm, *Pieris rapae* (L.) [190], and diamondback moth, *Plutella xylostella* (L.) [191]. This blackening takes two or more days to occur [187], so very recently parasitized eggs will probably not be detected using this method. Assessments of parasitism based strictly on proportion of black eggs will not provide information on species-level parasitism by *Trichogramma* unless there is good information on which species are present in the environment. A subsample of parasitized eggs may be used for rearing of adult *Trichogramma* for species-level identifications to obtain information on species present and their relative abundances [188]. Species-level identifications of *Trichogramma* are difficult and generally require examination of male genitalia, meaning mounting on slides and examination by a specialist will be necessary for reliable identifications.

Predation on crop pests can be difficult to detect, let alone estimate, but research on predation on crop pests has made great strides in recent years. Predation has been studied in a variety of ways, including video recording [192], use of sentinel prey [193], and use of markers and analysis of predators for prey remains, including molecular methods for analysis of predator gut contents [194]. Artificial caterpillars made of modeling clay have been used to assess predation in insecticide treated vs. untreated cotton plots in Uganda [195], and in vetch and barley cover crops in Italy [196]. This method allows identification of predators reliably at course taxonomic levels (bird, mammal, and arthropod) [197]. Approaches for assessing natural enemy impacts in agroecosystems have been reviewed recently [198,199].

## 4. Conclusions and Further Considerations

Beneficial arthropods comprise a wide diversity of taxa and life histories, and the methods available to sample them are accordingly diverse as well. Any sampling method will have inherent advantages and limitations (Table 1), and it is essential that researchers be aware of these so that results can be properly interpreted. Different sampling methods for the same taxonomic group will often yield different results. This means that consistency in methodology is essential for results to be comparable. It also means results of different sampling methods may be complimentary to an extent, and that use of multiple trapping methods may give more complete results than use of a single method. This is particularly important in studies focusing on diversity. Unfortunately, in the vast majority of cases we do not have information on the “true” faunal composition which could serve as a point of reference with which to compare our sampling methods. However, multiple sampling methods should increase completeness to the extent that the results of the different methods are nonoverlapping. More research is needed on development of novel sampling methods for beneficial arthropods and comparisons of the different methods.

Since most invertebrate monitoring programs rely heavily on lethal sampling, potential effects of monitoring on beneficial arthropod abundance and diversity are a concern. Only a few studies have specifically addressed effects of lethal sampling, and most of these focus on vertebrates, including shrews [200], lizards [201], and small mammals [202]. Among beneficial arthropods, potential impacts of lethal sampling on bees have been examined [203]. These studies have consistently found no evidence for long-term negative impacts on these communities. Even so, researchers should try to avoid sacrificing excessive numbers of specimens. In studies addressing beneficial arthropod diversity, a predetermined point beyond which sampling is unnecessary can be established based on estimates of species richness [179,204,205], although for many arthropod taxa a major challenge is the on-the-spot specimen identifications that would be required.

It is also important to note that many sampling methods will produce incidental captures, or “bycatch”. While this bycatch may be unimportant for the study at hand, it could contain specimens of concern for pest management, or of research or conservation interest. For instance, pitfall trap studies have yielded important information via incidental bee captures in coastal sage scrub habitats of southern California [206] and in the Palouse Prairie of eastern Washington and northwestern Idaho, USA [207]. Conversely, Malaise traps with a collection container at the base have been successfully used in conjunction with pitfall traps to sample carabids in seasonally flooded hardwood forest canopy gaps; use of pitfall traps alone would have resulted in a 38% reduction in species richness [208]. As these researchers note, flight is an important dispersal mechanism in many carabid species, and macropterous species are often predominant in disturbed habitats [209,210,211,212]. This suggests that Malaise traps or perhaps less expensive flight interception traps could be useful in augmenting pitfall trap collections of carabids in agroecosystems. Malaise traps can also be used to effectively sample spiders, as shown in a study of spider diversity in peatland and wet grassland across Ireland [213]. In this study, Malaise trap collections were to a great degree complimentary with pitfall trap collections. As these authors observe, Malaise traps may not be suitable as a sole method of sampling spiders, but traps in use to collect insects may also provide valuable data on the spider fauna. While the above studies were done in nonagricultural environments, they underscore the potential for bycatch or nontarget captures to contribute important and perhaps unexpected information. Bycatch not intended to be used can be made available to appropriate specialists to avoid waste [214].

It should also be noted that correct identification of arthropod specimens is often much more challenging and time-consuming than the sampling itself. Help from appropriate specialists is often needed, and it is wise to explicitly budget for identification services. Furthermore, collected specimens will require care and management. It is wise to construct a plan for specimen identification, data entry and organization, and collection management before sampling begins.

As habitat destruction, climate change, and other anthropogenically-driven changes continue to unfold, intensive agricultural practices will face new challenges. If climate change leads to increased insect pest populations and changes in pest geographic ranges, it will be difficult to resist the temptation to respond with even greater reliance on insecticides. However, such a response would likely contribute to the already high rate of biodiversity loss, including loss of beneficial arthropod diversity. An increased role for native pollinators and natural enemies in agroecosystems would be one strategy for decreasing reliance on insecticides. However our knowledge of these beneficial arthropods is only as good as the reliability of our sampling. Reliable sampling and monitoring methods for these beneficial arthropods will be essential for assessing the effects of environmental disturbances and effectiveness of conservation efforts. Hopefully this paper provides information that will prove useful in choosing appropriate methods for assessing beneficial arthropod health and effectiveness in agricultural systems.

## Figures and Tables

**Table 1:** Beneficial arthropod sampling methods included in this paper, focus, type of estimate, relative cost and labor, and comments on advantages and limitations of each method.

Method	Focus	Absolute or Relative Estimate	Cost	Labor	Comments
Bowl traps	Bees, wasps, flies	Relative	Low	Low	
Blue vane traps	Bees, wasps, flies	Relative	High	Low	Bulky to transport
					Potential oversampling of large bees
Trap-nests	Trap-nesting bees and wasps	Relative	Low	High	High selectivity
Targeted netting w/baits	Orchid bees	Relative	High	High	High selectivity
					Collector abilities can vary
Malaise traps	Flying insects	Relative	High	Low	High and diverse yield
					Significant setup time
					Potential damage due to wind or vandalism
Targeted netting	Flying insects; insects on vegetation	Relative	Low	High	High selectivity
					Collector abilities can vary
Sweep-netting	Arthropods on vegetation	Relative or absolute	Low	High	High and diverse yield
					See a past paper [143] for absolute estimates
Direct counts on vegetation	Arthropods on vegetation	Relative or absolute	Low	High	High selectivity
					Significant training may be required
Shaking/beating vegetation	Arthropods on vegetation	Relative or absolute	Low	High	May damage plants
Suction samplers	Plant and litter arthropods	Relative or absolute	High	High	High and diverse yield
Pitfall traps	Epigeic arthropods	Relative	Low	Low	Significant setup time
					High and diverse yield
					Sensitive to rainfall
					Potential animal disturbance
Emergence traps	Epigeic arthropods	Absolute	High	High	May affect microclimate
					Sampling excludes migrants
Soil flooding	Epigeic arthropods	Absolute	Low	High	
Microhabitat removal and extraction	Epigeic arthropods	Absolute	Low	High	
Cryptozoan boards	Epigeic arthropods	Relative	Low	High	Placement duration may affect yield
Sticky traps	Parasitic Hymenoptera	Relative	Low	Low	Baits increase expense
Estimates of parasitism	Parasitoids	Relative	Low	High	High selectivity
					Direct measure of natural enemy effects
Estimates of predation	Predators	Relative	Variable	High	Direct measure of predation
